# Clinicopathological Findings and Prognosis in Canine Cases Diagnosed As Primary Hypoplasia of the Portal Vein

**DOI:** 10.3389/fvets.2017.00224

**Published:** 2017-12-21

**Authors:** Makoto Akiyoshi, Masaharu Hisasue, Masami Akiyoshi

**Affiliations:** ^1^Laboratory of Veterinary Internal Medicine 2, School of Veterinary Medicine, Azabu University, Kanagawa, Japan; ^2^Akiyoshi Animal Clinic, Kanagawa, Japan

**Keywords:** dog, fibrinogen, liver, primary hypoplasia of the portal vein, prognosis, total bile acid concentrations

## Abstract

Canine primary hypoplasia of the portal vein (PHPV) is a microscopic malformation of the hepatic vasculature. The prevalence, clinical signs, and clinicopathological findings of PHPV in dogs are unclear, because there are few reports concerning PHPV in the veterinary literature. This retrospective study reviewed clinical records and liver biopsy data from 48 dogs with hepatic disease that were examined at a private veterinary hospital in Japan between April 2011 and March 2014 to determine the prevalence of PHPV among dogs that underwent liver biopsy and to determine the clinical and clinicopathological findings of PHPV in dogs. Records for all 48 dogs that underwent liver biopsy were investigated. Collected data included signalment, clinical signs, physical examination findings, complete blood cell count, chemistry results, pre-and postprandial serum total bile acid concentrations, coagulation profiles (prothrombin time, activated partial thromboplastin time, fibrinogen, and antithrombin), and abdominal ultrasonography findings at the first medical examination. The diagnosis of PHPV was made on the basis of histological examination of hepatic biopsy specimens and portography or CT angiography. Among the 48 canine cases, 28 dogs (58.3%) were diagnosed with PHPV, which was the most common diagnosis. The most frequent clinical sign in dogs with PHPV was asymptomatic persistently increased liver enzymes (57.1%). Toy poodles were at a significantly higher risk of PHPV than other breeds among dogs that underwent liver biopsy (*P* < 0.001). The median survival time of dogs with PHPV was more than 5 years. Plasma fibrinogen concentration below the reference range was an indicator of PHPV in this study. Dogs with PHPV frequently had mild clinical signs and a favorable prognosis.

## Introduction

Primary hypoplasia of the portal vein (PHPV) is a congenital vascular anomaly that occurs in dogs (1, 2). PHPV is characterized by abnormally small extrahepatic or intrahepatic portal veins, which result in diminished hepatic perfusion and the potential for portal hypertension (1, 2). Portal hypertension, a persistently high pressure in the portal vein, may result from a wide variety of diseases and frequently leads to the formation of acquired portosystemic collaterals (APSCs) (3).

Histologically, PHPV has a typical pattern of portal vein hypoperfusion, small or absent portal veins, proliferation of hepatic arterioles, and hepatocyte atrophy (1, 2). The diagnosis of PHPV is made with histological examination of wedge biopsies of the liver in combination with ultrasonographic findings and portography or CT angiography to exclude the presence of macroscopic portosystemic shunt (PSS), arteriovenous fistula, or portal vein thrombosis (4–8). Previous studies have used several terms to identify this congenital condition, including microvascular dysplasia, non-cirrhotic portal hypertension, and hepatoportal fibrosis (1, 2, 9–12). In 2006, PHPV was proposed by the World Small Animal Veterinary Association Liver Standardization Group (1) as a single term for all of these conditions because there are no clinical or biochemical findings to suggest that these diseases are different from PHPV. However, the pathological status varies, with mild conditions recognized as persistent or intermittent slight increases in liver enzymes, including alanine aminotransferase (ALT), alkaline phosphatase (ALP), aspartate aminotransferase (AST), and γ-glutamyl transferase (GGT). By contrast, severe cases may present with neurologic signs, gastrointestinal tract signs, and lethargy (9, 10, 12–14).

The first prevalence survey on canine and feline liver diseases at a secondary veterinary hospital in Japan was recently performed (11). That study demonstrated that the most common canine liver disease in liver biopsy samples was congenital hepatic vascular malformation, including PSS and PHPV. However, epidemiological studies on canine PHPV at private veterinary hospitals in Japan have not been reported. In addition, the clinical signs and clinicopathological findings of PHPV are unclear, as there have been few reports on the condition (3, 9). Dogs with PHPV should be identified at an early age to avoid confusion in future diagnostic evaluations of other primary hepatic diseases. The purpose of this study was to investigate the prevalence of PHPV in dogs at a private veterinary hospital in Japan and to identify clinicopathological findings in dogs with PHPV.

## Materials and Methods

All records for dogs that underwent liver biopsy at one private veterinary hospital from April 2011 to March 2014 were reviewed. The search revealed 52 cases. All 52 dogs were suspected of liver disease because of persistently increased liver enzymes for over 2 months and total bile acid concentrations (TBA) above the reference range, or because of abnormal findings in the liver parenchyma on ultrasonography, including target or mass lesions. All liver biopsies were performed as wedge biopsies at open surgery in dogs suspected of having liver disease. All dogs had single-wedge biopsy specimens collected from more than three liver lobes. TBA evaluation (pre- and postprandial) was performed several times in dogs with persistently increased liver enzymes; dogs with TBA above the reference range underwent liver biopsy.

Inclusion criteria were primary hepatic disease without concurrent disease. Forty-eight dogs met these criteria and were included in this study.

Criteria for the diagnosis of PHPV included histological findings on hepatic biopsy as well as portography or CT angiography to rule out PSS, based on previous reports (1, 9, 15–17). Wedge liver samples were fixed in 10% neutral-buffered formalin, embedded in paraffin, sectioned at 4 µm thickness, and stained with hematoxylin and eosin before microscopic evaluation by a board-certified veterinary pathologist.

**Figure 1:**
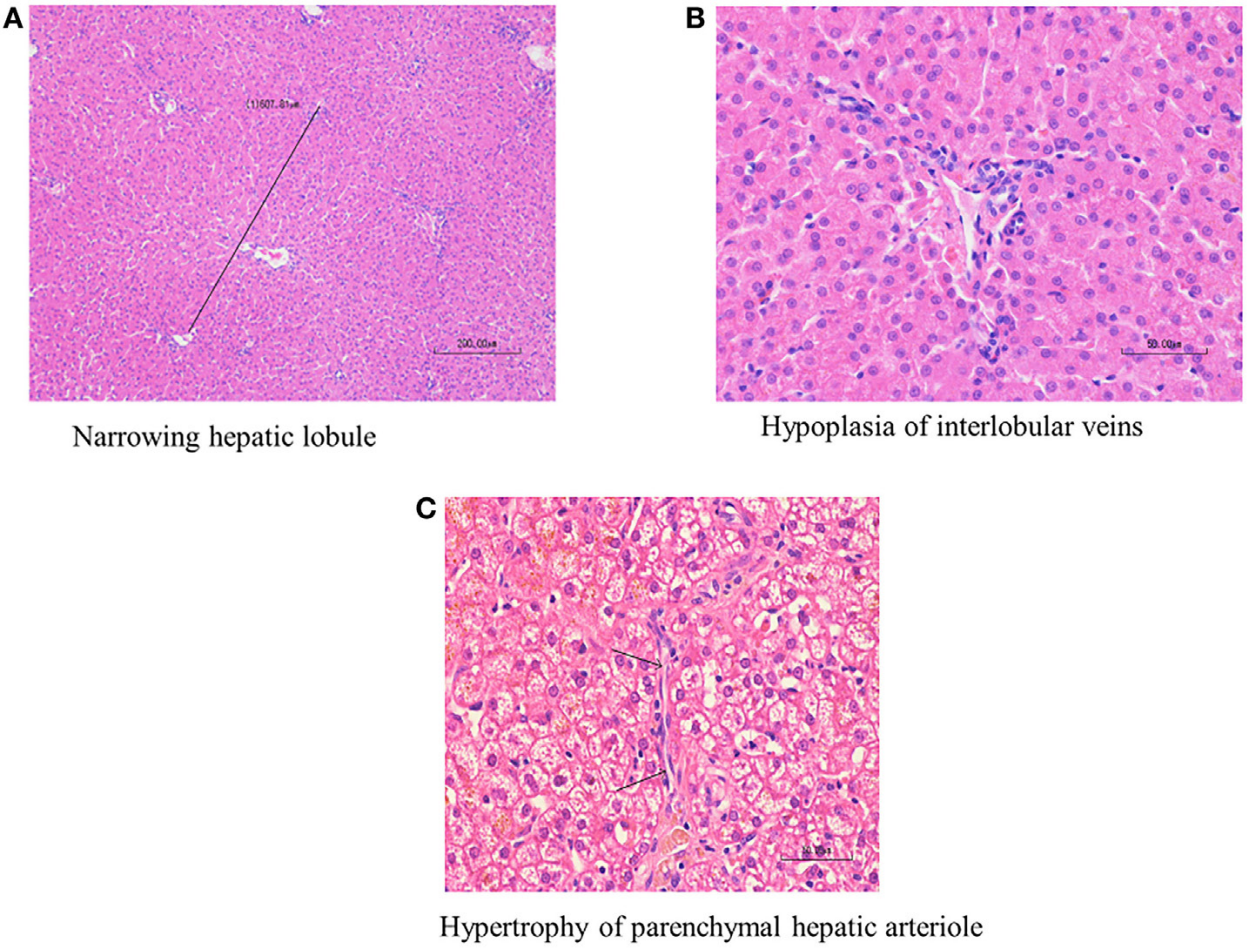
Histological findings of primary hypoplasia of the portal vein in the liver. Hypoplasia of the portal vein, narrowed hepatic lobule, bile duct proliferation, and arteriolar dilation. Marked diminution of the portal vasculature and mild arteriolar hyperplasia are noted in the interlobular connective tissue. **(A)** Narrowed hepatic lobule (medium magnification, 400×). **(B)** Hypoplasia of interlobular veins. The arrow indicates hypertrophy of parenchymal hepatic arteriole (high magnification, 1,000×). **(C)** The arrow indicates hypertrophy of parenchymal hepatic arteriole (high magnification 1,000×).

Histological diagnosis of PHPV was made according to the following published criteria (12, 15): hepatocellular atrophy, diminution of the portal vasculature with/without portal arteriolar hyperplasia, or hypertrophy of parenchymal hepatic arterioles. Hepatocellular atrophy was evaluated both according to hepatic lobular diameter and subjective impression of the size of individual hepatocytes (Figures 1A–C). Board-certified veterinary pathologists have confirmed that hepatic lobular diameter in dogs with no hepatic vascular anomaly usually falls in the range between 1.5 and 2 mm, based on repeated measurements of 25 normal liver samples from autopsy and biopsy cases (unpublished data collected at a private laboratory by board-certified veterinary pathologist Mitsui Ikki).

CBCs were measured with Celltacα (Nihon Kohden, Tokyo, Japan). Blood chemistry panels were performed with Fuji DRI-CHEM 7000V (FUJI FILM, Tokyo, Japan). Serum TBA and blood coagulation tests [prothrombin time (PT), activated partial thromboplastin time (APTT), fibrinogen, and antithrombin (AT)] were performed by commercial laboratories (IDEXX Japan, Tokyo, Japan and Hoken Kagaku, Kanagawa, Japan, respectively). Preprandial and postprandial TBA were measured with the enzyme cycling method using a Beckman Coulter AU5800. Blood coagulation analysis was performed on plasma. PT was measured with the quick method, APTT with the ellagic acid activation method, fibrinogen with the thrombin time method, and AT with a synthetic chromogenic substrate method. The following parameters were investigated: white blood cell count, hemoglobin, platelet count, blood urea nitrogen, ALT, AST, ALP, GGT, total bilirubin (T-Bil), total protein, albumin, ammonia (NH_3_), glucose, total cholesterol, TBA, PT, APTT, Fib, and AT. Levels above the reference range were considered abnormal for white blood cell count, ALT, AST, ALP, GGT, T-BiL, NH_3_, TBA, PT, and APTT. Levels below the reference range were considered abnormal for hemoglobin, platelet count, blood urea nitrogen, total protein, albumin, glucose, total cholesterol, Fib, and AT. Abdominal ultrasonography was performed in all dogs. The existence of a mass or target lesions in the liver parenchyma and the existence of PSS or APSCs were investigated thoroughly on the basis of ultrasonography.

Forty-eight of the 52 dogs had primary liver disease without concurrent disease, according to physical examination and ultrasonogaphy. The remaining four cases had complicated diseases including splenic tumor (two cases) and gastrointestinal tumor (two cases). These four cases were excluded from this study because the primary cause of disease was not in the liver. The medical records of the 48 dogs were reviewed. Based on histological examination, dogs were classified into two groups: the PHPV group and the non-PHPV group.

Data from the 48 dogs, including signalment, clinical signs, CBC results, chemistry results, pre- and postprandial TBA results, coagulation profiles and abdominal ultrasonography at the first medical examination, and results of histological examination of hepatic biopsy specimens, were reviewed. The medical records of all 48 dogs were complete.

Statistical analysis was performed with SPSS Statistics 22 (IBM Japan, Ltd.). The sample size was determined using a level of significance of 0.05, a power of 0.80, and an effect size of 1.0. According to these values, more than 17 data points were needed in each group; the number of cases in this study met the sample size requirements. For statistical analysis, Fisher’s exact test was performed to evaluate sex and breed differences. The Mann–Whitney *U* test, single variable logistics analysis, and multivariate logistics analysis were performed to evaluate age, white blood cell count, hemoglobin, platelet count, blood urea nitrogen, ALT, AST, ALP, GGT, T-Bil, total protein, albumin, NH_3_, glucose, total cholesterol, pre- and postprandial TBA, PT, APTT, fibrinogen, and AT. Based on results of single variable logistics analysis, multivariate logistics analysis was performed with choice of the explanatory variable by the variable increase method using the AIC. *P* < 0.05 was considered statistically significant. The day of histological diagnosis was defined as the start date for the observation period; median survival time was investigated with Kaplan–Meier curves.

## Results

Primary hypoplasia of the portal vein was the most frequent histological diagnosis, present in 28 of the 48 dogs (58.3%; PHPV group). Diagnoses in the remaining 20 dogs (non-PHPV group: 20/48; 41.7%) included neoplasia (7/48; 14.6%), inflammatory disease including hepatitis and cholangitis (6/48; 12.5%), degeneration (4/48; 8.3%), PSS (2/48; 4.2%), and cirrhosis (1/48; 2.1%). In the PHPV group, central venous dilation, hypertrophy, and perivascular fibrosis were sometimes observed. Hepatocytes showed various degrees of degeneration, with the majority of cases being low-to-intermediate degree. Histological changes suggestive of neoplasm, infection, thrombosis, or inflammation were not observed in the PHPV group.

Clinical signs in the PHPV group included gastrointestinal tract signs (9/28; 32.1%), including vomiting, diarrhea, and anorexia; neurologic signs (3/28; 10.7%), including seizures, ataxia, and shivering; and lethargy (2/28; 7.1%). Of the 28 dogs in the PHPV group, two presented with both neurological and gastrointestinal signs. Sixteen of the 28 dogs had no clinical signs other than persistently elevated liver enzymes. In these 16 cases, increased liver enzymes were detected on routine medical examination or on preoperative blood tests for castration/spay. All 28 dogs in the PHPV group had persistently increased liver enzymes for over 2 months.

Clinical signs in the non-PHPV group included gastrointestinal tract signs (11/20; 55%), including vomiting, diarrhea, and anorexia; neurologic signs (4/20; 20%), including seizures, ataxia, shivering, and lethargy (3/20; 15%); and asymptomatic persistently increased liver enzymes (2/20; 10%).

Abdominal ultrasonography was performed in all 48 dogs. Twenty-three dogs in the PHPV group had no abnormal findings of the liver parenchyma or hepatic blood vessels. The remaining five dogs had a small amount of ascites and APSCs. In all five of these dogs, a dilated left testicular or ovarian vein was seen. Abdominal findings in the non-PHPV group were as follows: mass or target lesion of the hepatic parenchyma (9/20; 45%), PSS (2/20; 10%), and small amount of ascites with APSCs (2/20; 10%). Seven dogs in the non-PHPV group had no abnormal findings of the liver parenchyma or hepatic blood vessels on ultrasound exam. Portography was performed in 28 dogs at the time of exploratory surgery (25 dogs in the PHPV group and three dogs in the non-PHPV group). CT angiography was performed in five dogs before liver biopsy (three dogs in the PHPV group and two dogs in the non-PHPV group).

The mean age of the 28 dogs in the PHPV group was 3.72 ± 3.73 years (median = 2.85 years; range = 0.3–13 years) (Table 1). Eleven dogs (39.3%) were younger than 1 year. The mean age of the 20 dogs in the non-PHPV group was 8.4 ± 3.59 years (median = 8.15 years; range = 2–14 years) (Table 1). No sex prevalence was found (PHPV group: 13 males, 15 females; non-PHPV group: 8 males, 12 females) (Table 2). The breeds in the PHPV group included toy poodle (eight cases: four males and four females), miniature dachshund (six cases: three males and three females), Chihuahua (three cases: two males and one female), Yorkshire terrier (two cases: both males), papillon (two cases: both females), miniature schnauzer (one case: male), shih tzu (one case: female), Maltese (one case: male), Shiba (one case: female), flat-coated retriever (one case: female), golden retriever (one case: female), and American cocker spaniel (one case: female) (Table 2). The breeds in the non-PHPV group included miniature dachshund (seven cases: five males and two females), French bulldog (two cases: one male and one female), miniature schnauzer (two cases: both female), Welsh corgi (two cases: both female), Irish setter (one case: female), Italian greyhound (one case: female), shih tzu (one case: male), Maltese (one case: female), Pomeranian (one case: female), Shiba (one case: male), and mixed breed (one case: female) (Table 2).

**Table 1:** Ages, clinicopathological results of the 48 dogs of the non-primary hypoplasia of the portal vein (PHPV) group and the PHPV group when they were first examined.

	non-PHPV					PHPV				
	Reference range	Number	Abnormal rate	Median	Range	Number	Abnormal rate	Median	Range	*P*-value
Age	–	20	–	8.150	2.000–14.000	28	–	2.850	0.300–13.000	<0.001*
WBC (/μl)	5,000–17,000	20	7 (35%)	10,350.000	5,200.000–90,000.000	28	5 (17.9%)	11,900.000	9,400.000–42,000.000	0.084
Hb (g/dl)	12.0–18.0	20	5 (25%)	17.200	5.600–21.600	28	0 (0%)	17.150	12.500–22.800	0.676
PLT (10^4^/μ)	18.0–50.0	20	3 (15%)	31.800	4.600–79.700	28	0 (0%)	32.350	19.000–63.100	0.558
BUN (mg/dl)	9.2–29.2	20	2 (10%)	18.650	3.900–111.000	28	3 (10.7%)	17.350	8.200–39.500	0.341
Alanine aminotransferase (U/l)	17–78	20	15 (75%)	133.500	21.000–879.000	28	13 (46.4%)	67.500	27.000–324.000	0.011*
Aspartate aminotransferase (U/l)	17–44	20	8 (40%)	36.500	15.000–146.000	28	3 (10.7%)	31.000	16.000–87.000	0.286
Alkaline phosphatase (U/l)	47–254	20	12 (60%)	253.500	61.000–10,500.000	28	21 (75%)	429.000	77.000–3,500.000	0.517
γ-Glutamyl transferase (U/l)	5.0–14.0	20	9 (45%)	14.000	8.000–20.000	28	11 (39.3%)	12.000	6.000–22.000	0.423
T-Bil mg/dl	0.1–0.5	20	2 (10%)	0.400	0.010–1.700	28	0 (0%)	0.400	0.300–0.800	0.508
TP (g/dl)	5.0–7.2	20	1 (5%)	7.300	2.800–9.600	28	0 (0%)	6.650	5.200–10.600	0.285
Alb (g/dl)	2.6–4.0	20	3(15%)	3.450	1.300–4.700	28	2(7.1%)	3.500	1.800–4.400	0.908
NH3 (μg/dl)	16–75	20	6 (30%)	34.000	15.000–324.000	28	4(14.3%)	30.000	18.000–190.000	0.245
Glu (mg/dl)	75–128	20	4 (20%)	101.000	43.000–211.000	28	1(3.6%)	99.000	68.000–120.000	0.826
TCHO (mg/dl)	111–312	20	2 (10%)	230.500	84.000–454.000	28	1 (3.6%)	221.000	114.000–358.000	0.653
Pre total bile acid concentrations (TBA) (μmol/l)	<10	20	12 (60%)	15.300	1.500–611.400	28	8 (28.6%)	8.050	1.000–182.200	0.038*
Post TBA (μmol/l)	<15	20	15 (75%)	30.900	3.000–850.000	28	14 (50%)	15.150	1.000–258.000	0.074
Prothrombin time (s)	6.0–9.0	20	7 (35%)	8.550	6.800–120.000	28	11 (39.3%)	8.800	6.500–11.700	0.645
Activated partial thromboplastin time (s)	13.0–19.0	20	7 (35%)	15.000	10.600–250.000	28	3 (10.7%)	15.000	11.200–32.600	0.242
Fibrinogen (mg/dl)	160–400	20	5 (25%)	193.500	25.000–864.000	28	20 (71.4%)	128.000	63.000–527.000	0.015*
Antithrombin (%)	95–135	20	9 (45%)	97.500	59.600–140.000	28	6 (21.4%)	106.000	49.000–140.000	0.402

*The asterisk shows significant difference (*P* < 0.05)*.

**Table 2:** Gender, breeds, and numbers of the 48 dogs of the non-primary hypoplasia of the portal vein (PHPV) group and the PHPV group when they were first examined.

	Non-PHPV			PHPV		
Group	20			28		
Gender	20			28		
Male		8	40.00%		12	42.86%
Female		12	60.00%		16	57.14%
Breed	20			28		
Miniature schnauzer		2	10.00%		1	3.57%
Miniature dachshund		7	35.00%		6	21.43%
Irish setter		1	5.00%		0	0.00%
American cocker spaniel		0	0.00%		1	3.57%
Italian greyhound		1	5.00%		0	0.00%
Welsh corgi		2	10.00%		0	0.00%
Golden retriever		0	0.00%		1	3.57%
Shih tzu		1	5.00%		1	3.57%
Chihuahua		0	0.00%		3	10.71%
Toy poodle		0	0.00%		8	28.57%*
Papillon		0	0.00%		2	7.14%
Flat-coated retriever		0	0.00%		1	3.57%
French bulldog		2	10.00%		0	0.00%
Pomeranian		1	5.00%		0	0.00%
Maltese		1	5.00%		1	3.57%
Yorkshire terrier		0	0.00%		2	7.14%
Mixed breed dog		1	5.00%		0	0.00%
Shiba		1	5.00%		1	3.57%

*The asterisk shows significant difference (*P* < 0.05)*.

According to laboratory reference intervals (Table 1), the most common clinicopathologic abnormality in the PHPV group was an increase in one or more liver enzymes, including ALT, AST, ALP, and GGT (22/28 cases; 78.6%). Increased ALT was seen in 13 of 28 dogs (46.4%). Increased ALP was seen in 21 of 28 dogs (75.0%) and was recognized as the most frequent abnormality. AST level was high in three cases (10.7%). Abnormal GGT values were observed in 11 cases (39.3%). Increased NH_3_ concentration in the peripheral blood was seen in four cases (14.3%). Other abnormalities had limited or no occurrence, including increased T-Bil (0 cases; 0%) and decreased blood urea nitrogen (three cases; 10.7%), albumin (two cases; 7.1%), glucose (one case; 3.6%), and total cholesterol (one case; 3.6%) (Table 1). Among the 20 dogs in the non-PHPV group, the most common abnormality was increased ALT (15/20; 75%). Increased AST was seen in 8 of the 20 dogs (40%), increased ALP in 12 (60%), and increased GGT in nine (45%). Increased NH_3_ was seen in 6 of the 20 dogs (30%) and increased T-Bil in 2 (10%).

In the PHPV group, increased TBA (preprandial TBA >10 μmol/l and/or postprandial TBA >15 µmol/l) during food deprivation, 2 h after feeding, or both was seen in 15 of 28 dogs (53.6%). In the non-PHPV group, increased TBA during food deprivation, 2 h after feeding, or both was seen in 16 of 20 dogs (80%). In the blood coagulation analysis, the most common abnormality in the PHPV group was decreased fibrinogen (<160 mg/dl; reference range: 160–400 mg/dl), which was found in 20 of 28 dogs (71.4%). In the PHPV group, 96.4% of dogs had either a low fibrinogen or elevated TBA; combining these tests increased the diagnostic sensitivity (Table 3). Prolonged PT was seen in 11 of 28 dogs (39.3%), prolonged APTT was seen in 3 (10.7%), and decreased AT activity was seen in 6 (21.4%) (Table 1). In the non-PHPV group, prolonged PT was seen in seven of 20 dogs (35%), prolonged APTT in 7 (35%), decreased fibrinogen in 5 (25%), and decreased AT activity in 9 (45%).

**Table 3:** The association of total bile acid concentrations (TBA) and fibrinogen levels of the primary hypoplasia of the portal vein (PHPV) group.

	PHPV (28 cases)
Increase of TBA levels Decrease of fibrinogen levels	15 (53.6%) 20 (71.4%)
1. Increase of TBA without fibrinogen abnormality 2. Decrease of fibrinogen without TBA abnormality 3. Increase of TBA and decrease of fibrinogen levels 4. Normal TBA and fibrinogen levels	5 (17.9%) 12 (42.9%) 10 (35.7%) 1 (3.57%)
Increase of TBA or decrease of fibrinogen	27 (96.4%)

According to Fisher’s exact test, the toy poodle breed was significantly overrepresented in the PHPV group (*P* < 0.001) (Table 2). The Mann–Whitney *U* test revealed that the PHPV group had a younger median age (*P* < 0.001), lower ALT (*P* = 0.011), lower preprandial TBA (*P* = 0.038), and lower fibrinogen (*P* = 0.015) than the non-PHPV group (Table 1). Based on the results of single variable logistics analysis, multivariate logistics analysis was performed with choice of the explanatory variable according to the variable increase method using the AIC. Multivariate logistics analysis revealed a significant difference in age between the groups (*P* = 0.004). ALT values and fibrinogen concentrations in the PHPV group were lower than those in the non-PHPV group; these differences were close to significance (Table 4).

**Table 4:** Results of logistics analysis.

	Single variable logistics regression analysis			Multivariate logistics regression analysis		
	OR	95% CI	*P*-value	OR	95% CI	*P*-value
Gender	0.889	0.277–2.854	0.843			
Age	0.732	0.608–0.881	0.001*	0.744	0.607–0.912	0.004
WBC	1.000	1.000–1.000	0.485			
Hb	1.102	0.946–1.284	0.213			
PLT	1.011	0.964–1.059	0.657			
BUN	0.957	0.912–1.004	0.072			
Alanine aminotransferase	0.992	0.984–0.999	0.032*	0.991	0.981–1.000	0.061
Aspartate aminotransferase	0.977	0.952–1.003	0.088			
Alkaline phosphatase	1.000	0.999–1.000	0.246			
γ-Glutamyl transferase	0.952	0.833–1.088	0.470			
T-Bil	0.802	0.067–9.595	0.862			
TP	0.906	0.596–1.377	0.644			
Alb	1.046	0.435–2.517	0.920			
NH_3_	0.994	0.982–1.006	0.297			
Glu	0.994	0.971–1.018	0.643			
TCHO	0.997	0.990–1.004	0.419			
TBA.Pre.	0.993	0.984–1.003	0.174			
TBA.Post.	0.995	0.988–1.003	0.208			
Prothrombin time	0.895	0.735–1.089	0.267			
Activated partial thromboplastin time	0.953	0.873–1.040	0.283			
Fibrinogen	0.995	0.990–1.000	0.061*	0.995	0.988–1.001	0.120
Antithrombin	1.012	0.986–1.039	0.381			

*The asterisk shows significant difference (*P* < 0.05)*.

**Figure 2:**
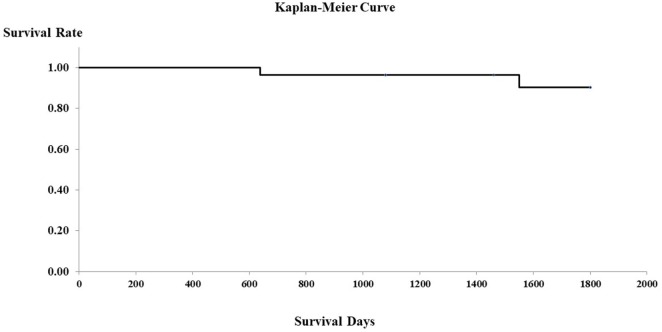
Survival curves of 28 dogs with primary hypoplasia of the portal vein (PHPV) (Kaplan–Meier). Surviving cases and dogs that died of disorders other than PHPV are censored (on the survival curves).

In the PHPV group, all 16 dogs without clinical signs other than increased liver enzymes were in good health for at least 5 years after the time of diagnosis. The seven dogs that presented with GI signs alone were in good health for at least 3 years after diagnosis. Of the two dogs with both neurological signs and GI signs, one died 639 days after diagnosis and one 1,550 days after diagnosis, as a result of hypoalbuminemia and hypoglycemia due to APSCs. The one dog with only neurological signs was in good health for at least 3 years after the time of diagnosis. The two dogs presenting with lethargy were in good condition for at least 1,460 days after the time of diagnosis. Figure 2 shows the Kaplan–Meier curve of the PHPV group. Mean survival time in the PHPV group was more than 5 years. The two dogs that died had APSCs at the time of diagnosis. In this study, there were differences in histopathological findings between PHPV with versus without APSCs.

## Discussion

Small breeds were most commonly affected with PHPV in this study, with toy poodles the predominant breed. This breed distribution agrees with that previously reported for PHPV (10, 11). Hirose et al. reported that PHPV, described as microvascular dysplasia, was often seen in Yorkshire terriers, toy poodles, and papillons (11). The reason that toy poodles were overrepresented in this study may be that for decades this dog breed was the most popular in Japan.

Portosystemic shunt was diagnosed in only two cases in this study, for a prevalence of 3.8%. The two dogs with PSS did not have concurrent PHPV. This 3.8% is a reasonable true prevalence for PSS at a private veterinary hospital, because no dogs would undergo surgery for PSS without liver biopsy. This study had two findings that differed significantly from past studies (11). First, the prevalence of vascular anomaly, including PHPV and PSS, was 62.5% in our study, which is more than twice the prevalence of 29.4% reported in a previous study (11). Second, 16 of 28 cases (57.1%) in the PHPV group were asymptomatic and those with clinical signs maintained good health with medications and diet modification. We hypothesize that most PHPV cases are asymptomatic or mild, and that other veterinarians have not performed diagnostics for PHPV. Therefore, it is possible that we might have investigated latent or early-stage PHPV cases. In the studies of Allen et al. (9) and van den Ingh et al. (3), the patients showed clinical abnormalities or severe illness. The two differences in findings may have resulted from selection bias between this and previous studies. Our findings demonstrate that PHPV is common among young asymptomatic dogs.

On clinicopathological examination, increased ALT (13/28; 46.4%) and TBA (15/28; 53.6%) and decreased fibrinogen (20/28; 71.4%) were seen. Multivariate analysis comparing the non-PHPV and PHPV groups revealed a significant difference in age between the groups (*P* = 0.004); differences in ALT (*P* = 0.061) and fibrinogen (*P* = 0.120) approached significance. The hypothesized reason for the difference in age between the groups was that the increase in liver enzymes was detected in routine medical examination or preoperative blood test for castration/spay. ALT levels may have differed because hepatocellular obstruction and swelling were less severe in the PHPV group than in the non-PHPV group, which included dogs with hepatitis and hepatic tumors. As the lesions of PHPV progress, ALT may increase. In this study, dogs with PHPV with APSCs had ALT levels that were much higher than the reference range. In the previous reports by Schermerhorn et al. (12) and van den Ingh et al. (3), severe cases of PHPV also had higher ALT.

Only half of our PHPV cases (15/28; 53.6%) had increased TBA, a percentage lower than that in previous reports (42/45; 93%) (18). The reason for increased TBA has been described by Allen et al. (9) as follows: hepatoportal blood flow is important in the delivery of hepatotrophic factors and in the filtration of enteric toxins. Partial microscopic shunting of blood allows a small fraction to bypass the liver, allowing enteric toxins to be released into the systemic circulation. The small fraction of shunted blood explains the less severe clinical signs and differences in TBA concentrations in dogs with PHPV compared with dogs with PSS. Interestingly, the TBA levels of the PHPV group in our study were lower than those reported in other studies (9, 12). TBA concentrations in PHPV might increase with age and might be related to clinical signs. The age at the time of diagnosis of PHPV is likely related to clinical signs, and there were 11 dogs under 1 year of age in the PHPV group in this study. 15 of 28 dogs (53.6%) in the PHPV group had pre- and postprandial TBA values above the reference range. However, all 28 dogs in the PHPV group eventually had increased TBA after several examinations. There may be two reasons for this finding. First, the degree of PHPV varied, and TBA increased above the reference range as PHPV progressed. This explanation was suspected based on the timeline, and there were few other causes for progressive increases in TBA. Second, TBA analysis is used as a function test for many liver diseases, and there are many secondary factors potentially influencing the results, such as digestive and absorption ability of the gastrointestinal tract and gallbladder status variability according to the day; hence, a consistent value is not obtained (19–21). Therefore, TBA testing should be performed several times, and should be used for diagnosis of PHPV only after careful exclusion of other liver diseases.

Fibrinogen concentrations in the PHPV group in this study were lower than the reference range. This may be due to the fact that the PHPV cases in our study (12 with clinical signs, 16 without clinical signs) were mostly asymptomatic compared with the 17 cases in the study of Allen et al. (16 with clinical signs, one without clinical signs). Dogs in the PHPV group with clinical signs might have had increased fibrinogen concentrations because of inflammatory lesions. In fact, fibrinogen concentrations among the 16 dogs without clinical signs in this study were lower than those among the 12 with clinical signs. Fibrinogen is the first blood coagulation factor and is an important factor in the stabilization of fibrin at the final stage of coagulation. Fibrinogen production in the liver decreases when a liver deficiency disorder is present (22–24). However, fibrinogen is an acute phase substance that increases in cases of severe inflammation and in neoplastic diseases of various organs (22–24). Therefore, compared with levels in patients with hepatic tumors or hepatitis, fibrinogen levels among dogs in the PHPV group that did not have inflammation were lower than the reference range. When fibrinogen is used to screen for PHPV, inflammation and tumors should be excluded with white blood cell counts and C-reactive protein levels (25, 26). In addition, if disseminated intravascular coagulation is suspected, it should be evaluated with fibrin/fibrinogen degradation products and D-dimer levels. In addition to fibrinogen, multiple coagulation factors are produced in the liver. PT and APTT do not necessarily quantitate factor activity levels. PT and APTT are determined by multiple factors, including fibrinogen. The only factor that was assayed individually in this study was fibrinogen. Therefore, the other factors might have been below reference ranges while PT and APTT remained within normal levels. We thought that some coagulation factors may be low level, and this abnormality is not affect the measurement of PT or APTT. However, the reason that only fibrinogen levels were low is unclear. Interestingly, the prevalence of elevated TBA was 53.6% (Table 3) and that of low fibrinogen was 71.4% (20/28). However, combining both tests increased the diagnostic sensitivity in the PHPV group (96.4%, 27/28). This finding suggests that combining these tests may be useful for detecting PHPV. However, improving sensitivity should help rule out PHPV.

The diagnosis of canine PHPV remains controversial among pathologists (1, 2, 9, 11, 15). Some pathologists propose that PHPV should be defined as microvascular dysplasia, because the lesions in the liver include not only PHPV but also microvascular proliferation. Therefore, in 2006, the Liver Standardization group of the World Small Animal Veterinary Association decided to abandon the name “microvascular dysplasia” in favor of PHPV, because the disease had already been reported as primary portal vein hypoplasia, which is a better description of the disease. Therefore, in this study, histological reports used the term PHPV, according to the Association’s definition. We have considered reports of microvascular dysplasia in past studies as PHPV in this study. These diagnostic terms will be unified in the future.

Survival time was more than 5 years in this study, indicating that the PHPV group had a favorable prognosis, similar to that previously reported (12). In the previous report by Schermerhorn et al., there was no clear long-term follow-up. Moreover, the report by Allen et al. did not include PHPV cases with APSCs (8). The prognosis for dogs with PHPV and APSCs may be bad; two dogs of five with APSCs died in this study. This prognosis was similar to that reported by van den Ingh et al. (14). In this study, it is unclear when APSCs develop in dogs with PHPV, because PHPV without APSCs did not progress to PHPV with APSCs. However, our findings suggest that PHPV, even if asymptomatic, requires early detection and periodic follow-up examinations, because PHPV is a congenital disease and may progress.

In conclusion, this study showed that toy poodles were overrepresented in PHPV and that fibrinogen level below the reference range was often seen in dogs with PHPV on routine examination, indicating that such analysis may help to detect canine PHPV. However, this study had the following three limitations: possible selection bias, small numbers, and retrospective design. Therefore, careful interpretation of the study results is necessary. The accumulation of further data from multiple primary veterinary hospitals in Japan and a prospective study will be necessary in the future.

## Ethics Statement

For this study using client-owned dogs, demonstrates a best practice of veterinary therapy and involves informed client consent. This study is retrospective study for investigation of diagnosis and prognosis, and the dosage of pharmaceutical products considered to be the animal experiment was not performed. The study protocol was approved by the animal committee of the Teaching Hospital of Azabu University.

## Author Contributions

MKA: study execution, data analysis, preparation manuscript, study design, drafting article, and final approval. MH: data analysis, study design, drafting article, and final approval. MSA: study execution, data analysis, study design, and final approval.

## Conflict of Interest Statement

The authors declare that the research was conducted in the absence of any commercial or financial relationships that could be construed as a potential conflict of interest.
